# Microstructure and Mechanical Properties of Tungsten Zircaloy-4 Diffusion Welding Interface

**DOI:** 10.3390/ma18122823

**Published:** 2025-06-16

**Authors:** Shaohong Wei, Yan Li, Ruiqiang Zhang, Bingfeng Wang, Tianjiao Liang, Wen Yin

**Affiliations:** 1 School of Materials Science and Engineering, Central South University, Changsha 410083, China; 2 Institute of High Energy Physics, Chinese Academy of Sciences (CAS), Beijing 100049, China; 3 Spallation Neutron Source Science Center, Dongguan 523803, China; 4 University of Chinese Academy of Sciences, Beijing 100049, China

**Keywords:** diffusing welding, hot isostatic pressure, spallation neutron source, target, Zircaloy-4 clad tungsten

## Abstract

The tungsten target block is widely used as a target material in spallation neutron sources. However, due to the poor corrosion resistance of tungsten, a corrosion-resistant metal layer needs to be coated on the surface. In this study, Zircaloy-4 coating on tungsten was prepared by hot isostatic pressure diffusion welding in the temperature range of 900 °C to 1400 °C. The microstructure and mechanical properties of the zirconium–tungsten interface were studied. The results show that a clear intermediate diffusion layer was formed at the interfaces, and no obvious defects were found. As the HIP temperature increased from 900 °C to 1400 °C, the thickness of the diffusion layer gradually increased from 0.28 μm to 10.74 μm. Composition and phase structure analysis of the intermediate diffusion layer showed that the main phase of the diffusion layer is ZrW_2_. The nanoindentation hardness results near the interface showed that the hardness of the ZrW_2_ diffusion layer was significantly higher than that of W and the zirconium alloy, reaching around 17.96 GPa. As the HIP temperature increased, the bonding strength between Zry-4 and W matrix first increased and then decreased, with the highest bonding strength of 83.9 MPa when the HIP temperature was 1000 °C.

## 1. Introduction

Spallation neutron sources are neutron factories that primarily utilize neutrons for fundamental research in physics, chemistry, and biology, such as ISIS [1], SNS [2], JSNS [3], and the China Spallation Neutron Source (CSNS) [4]. The CSNS is the first neutron scattering source in China, located in Dongguan City, Guangdong Province, in southern China. It produced its first neutron beam in October 2017 and was officially opened to users in 2018. At present, achievements have been made in materials, biology, chemistry, and other fields using CSNS neutron scattering experiments [5,6,7,8].

The target is the main equipment for producing neutrons in a scattering neutron source. High-energy protons bombard heavy metal materials, producing neutrons through fission reactions while releasing heat [9,10,11]. Generally, when the proton beam power is within one megawatt, the target material is mainly composed of solid metals with high atomic numbers, such as tungsten (W), tantalum (Ta), and uranium (U) [12,13]. This is because solid materials have a higher density and can achieve a high neutron yield. When the proton beam power is above megawatts, liquid target materials such as mercury are generally used, due to the increased thermal power density within the target. As power increases, it becomes more difficult for the target to dissipate heat. Liquid target materials can remove most of the heat through their own flow, thus solving the problem of heat dissipation [14,15].

Due to its high density, high melting point, and high thermal conductivity, W is used as a solid target material for scattering neutron sources. However, because of the poor corrosion resistance of W, it is necessary to coat it with a good corrosion-resistant layer to ensure the long-term use of the target material [16]. Ta metal is a good choice, and both have good applications in ISIS and the CSNS [17,18]. However, due to the high decay heat of Ta, a significant temperature increase may occur if the target loses cooling, resulting in irreversible consequences [19]. Decay heat refers to the thermal energy released by the radioactive decay of radioactive products and transuranic isotopes after a nuclear reactor is shut down. This heat arises from the kinetic energy of beta particles, gamma rays, and recoiling nuclei emitted during decay processes. It persists due to the residual radioactivity of short-lived and long-lived nuclides and gradually declines over time, governed by the decay constants of the radionuclides involved. Decay heat removal is critical for reactor safety, as its immediate post-shutdown intensity poses significant risks of core damage if cooling systems fail [20,21]. Therefore, the search for and preparation of low decay heat target plates are research directions for solid targets in neutron scattering sources. The CSNS has initiated a power upgrade project, and by the end of 2029, the power of the CSNS will reach 500 kW. Under high-power conditions, decay heat removal of the target under abnormal beam stop conditions will be a challenge.

Zircaloy-4 (Zry-4) is widely used as a cladding material for nuclear reactors due to its low neutron absorption rate, excellent high-temperature performance, and corrosion resistance [22,23]. Calculation results show that the decay heat of zirconium alloy is only one-tenth that of Ta. Using zirconium alloy to replace Ta as the target coating material can effectively reduce the decay heat of the target.

In this study, to develop solid target materials with low decay heat, Zry-4 alloy was diffusion-welded onto the surface of a W plate using the hot isostatic pressure (HIP) method. Microscopic analysis of the interface shows that after HIP diffusion welding, a clear diffusion layer is formed at the interface of W and Zry-4 alloy. The selected area electron diffraction pattern analysis results show that the intermediate diffusion layer is the ZrW_2_ phase. The nanoindentation hardness test results show that the hardness of the ZrW_2_ intermediate layer is significantly higher than that of the W and Zry-4 matrix. Meanwhile, as the HIP temperature increases, the thickness of the intermediate layer significantly increases, from 0.3 µm at 900 °C to 10.75 µm at 1400 °C.

## 2. Materials and Methods

**Figure 1:**
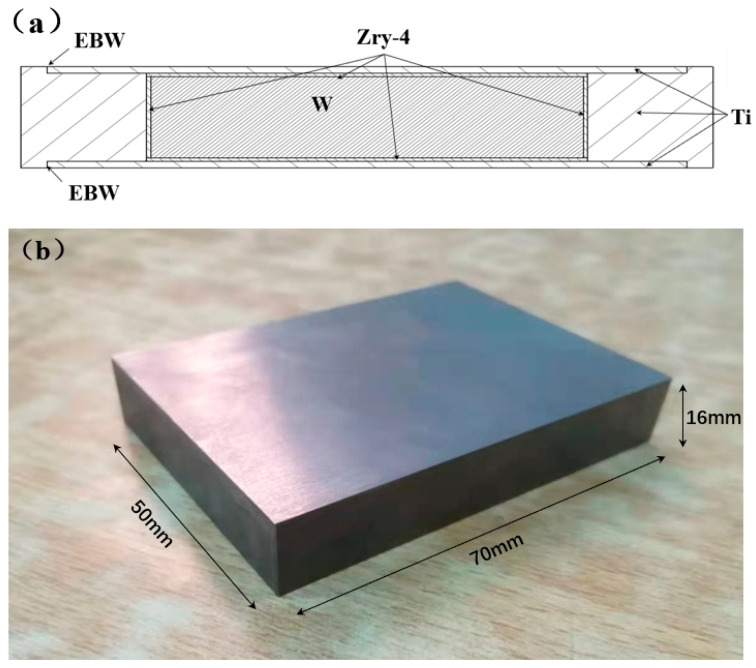
Preparation of W-coated Zry-4 target. (**a**) Assembly diagram before welding. (**b**) W-coated Zry-4 target block.

The tungsten rolled plates were procured from AT&M (Advanced Technology & Materials Co., Ltd. Located in Beijing, China), a Chinese manufacturer, featuring ultra-high purity with total trace impurities below 0.01% by weight. The elemental contaminants were specifically controlled at ≤10 ppm Fe, ≤10 ppm Ni, ≤10 ppm Si, ≤16 ppm C, ≤28 ppm O, and ≤10 ppm N in the tungsten matrix. Rolled Zirconium 4 alloy (Zry-4) plates were purchased from State Nuclear Bao Ti Zirconium Industry Co., Ltd. (China). The detailed chemical composition is as follows: Sn-1.3 wt%, Fe-0.21 wt%, Cr-0.11 wt%, Fe+Cr-0.32 wt%, O-0.13 wt%, and Zr-balance. Using a titanium alloy wrapper, the Zry-4 plate was fixed around the W block. The upper and lower surfaces of the titanium alloy wrapper were then welded using electron beam welding (EBW) in a vacuum, ensuring a tight fit between the Zry-4 block and the W block. After EBW sealing, the assembly was placed in a hot isostatic pressing furnace for diffusion welding under high temperature and high pressure. Finally, the wrapper was removed to obtain the Zry-4-coated tungsten target plate. Figure 1a shows the dimensions of the assembly after EBW and the W-coated Zry-4 target block after diffusion welding. Figure 1b shows the W target block after surface diffusion welding of the Zry-4 alloy. The thickness of the Zry-4 alloy cladding is 3 mm. Six different HIP processes were used for preparation, and the process parameters are shown in Table 1.

The mechanical tensile specimen features a sandwich structure, consisting of a 3 mm thick Zry-4 layer on the top and bottom surfaces, with a 10 mm thick W in the middle, and the parallel section has a width of 2 mm and a thickness of 1 mm. Mechanical characterization was performed using the following methods. Uniaxial tensile testing was conducted on an electromechanical universal testing machine (MTS Criterion 5 kN) with a controlled displacement rate of 1 mm/min. Nanoindentation analysis was performed using a load-controlled quasi-static nanoindentation protocol (Bruker HYSITRON TI980 system, manufacturer is Bruker, located in Billerica, MA, USA), employing a Berkovich diamond indenter [24]. The critical parameters were as follows: the peak load was 10 mN and the holding time was 2 s, the distance between two adjacent points was 3 μm, and the angle between the test line and the interface was 30°.

The specimen’s interface morphology and chemical compositions were obtained by a scanning electron microscope (ZEISS, Sigma500, Carl Zeiss AG, Located in Oberkochen, Baden-Württemberg, Germany) and electron probe micro-analysis (JXA-iHP200F, the manufacturer is JEOL Ltd., located in Tokyo, Japan). TEM lamella was prepared by Ga+ focused ion beam with a final beam energy of 2 kV. The microstructural analysis and elemental mapping of the diffusion zone were performed using an FEI Talos transmission electron microscope (TEM) equipped with energy-dispersive X-ray spectroscopy (EDX) capabilities.

## 3. Results

**Figure 2:**
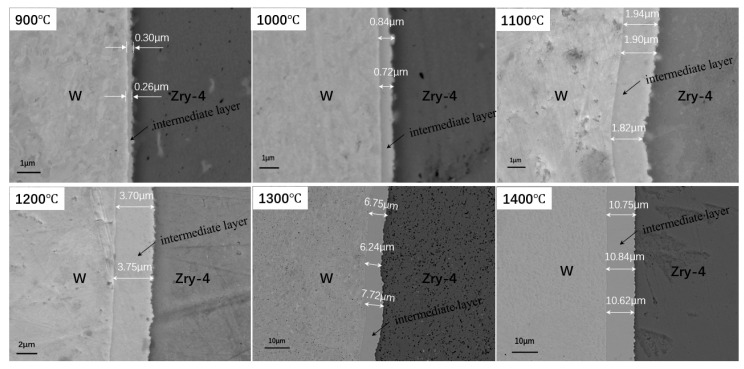
SEM images of sample interface at different HIP temperatures.

Figure 2 presents the SEM micrographs of the interface region for samples processed at different HIP temperatures. The images demonstrate that W and Zry-4 achieved complete metallurgical bonding at all investigated temperatures, exhibiting well-bonded interfaces free from observable defects such as pores or cracks. A distinct intermediate layer formed at the interface, for which the thickness showed a strong temperature dependence. Quantitative measurements revealed that the layer thickness increased from 0.28 μm at 900°C to 10.74 μm at 1400°C, representing significant growth with rising HIP temperature. The complete thickness data for all samples are compiled in Table 2.

The microstructure of the interface shows that the interface is relatively smooth between the intermediate layer and W, while the interface adjacent to the Zry-4 side exhibits significant undulation with a pronounced sawtooth morphology. This morphological difference likely stems from two factors: (1) the original W/Zry-4 interface retained surface irregularities from pre-welding preparation, and (2) zirconium’s relatively low melting point facilitated preferential diffusion from Zry-4 into the W matrix during HIP processing, ultimately forming the intermediate layer within the W side.

**Figure 3:**
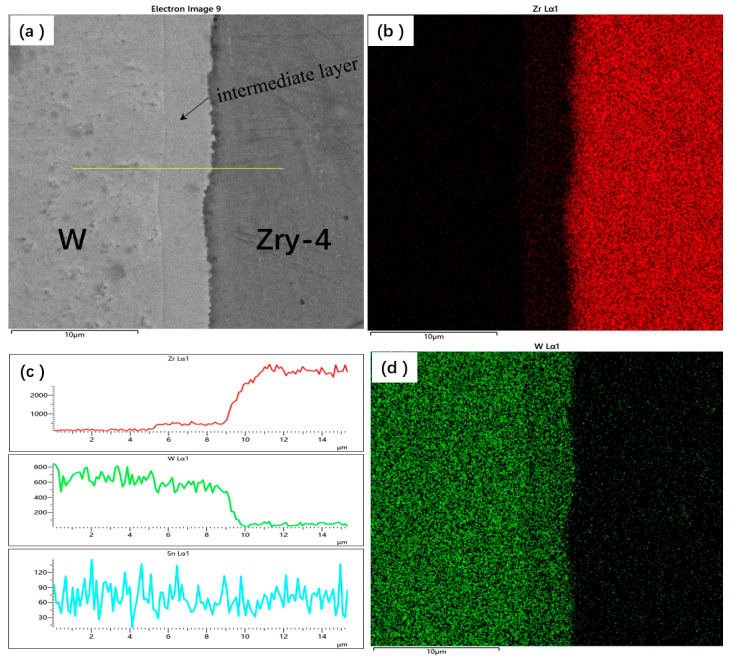
EDS results of the HIP-4 sample. (**a**) Surface morphology of the sample, (**b**) distribution of the Zr element content, (**c**) content curve of Zr and W elements at the interface, and (**d**) distribution of the W element content.

Figure 3 presents the X-ray energy-dispersive spectrum analysis (EDS) results for the HIP-4 sample. Figure 3b,d display the elements distribution of W and Zr across the interface; it can be clearly seen that a diffusion layer was formed in the middle of the interface that is composed of W and Zr elements. Figure 3c shows the line scanning profile across the interface, revealing an increasing Zr concentration gradient from left to right, accompanied by a corresponding decrease in the W content. A stable interfacial zone, approximately 3.7 mm thick, was observed at the intermediate interface, where the concentrations of both W and Zr elements remained essentially constant.

**Figure 4:**
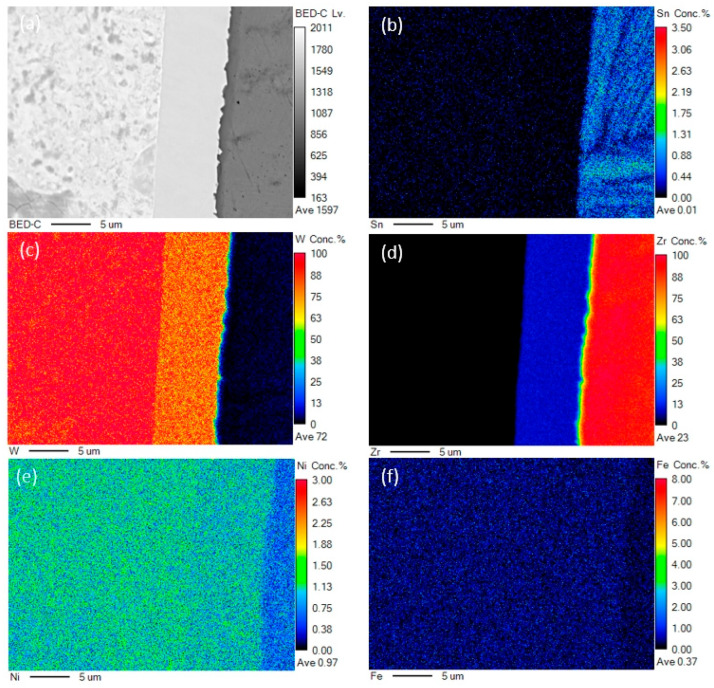
EPMA results of the HIP-6 sample. (**a**) Photo of sample interface. (**b**–**f**) Surface distribution of Sn, W, Zr, Ni, and Fe elements.

In order to characterize the interfacial elemental distribution in detail, electron probe micro-analysis (EPMA) was performed. Figure 4 shows the EPMA results for HIP6 samples at the interface region. Figure 4a is the backscattered electron photograph of the interface; it can be seen that the interface is well integrated, with no obvious pores or cracks. Figure 4b–f show the distribution and relative content of each element at the interface; the element maps clearly demonstrate pronounced concentration gradients for W, Zr, and Sn elements from the W side to the Zry-4 side, especially in the diffusion layer near the Zry-4 layer. Based on the elemental distribution analysis, the original interface position can be identified at the boundary between the diffusion layer and the Zry-4 alloy. During the diffusion process, Zr atoms diffuse into the W matrix and slowly accumulate at the W-Zry-4 interface, ultimately forming a stable W-Zr diffusion layer.

**Figure 5:**
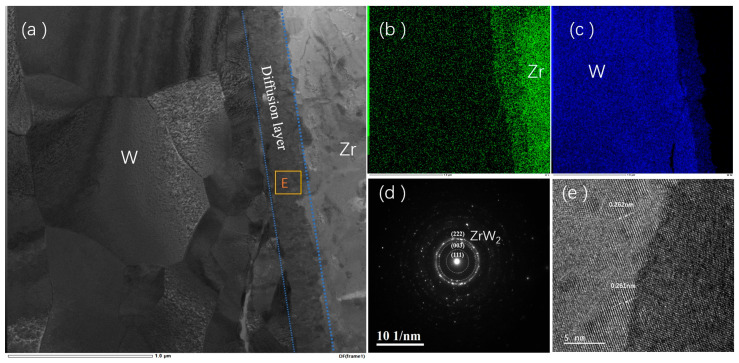
TEM results of the interface of HIP-1 sample, E is selecting the electron diffraction region. (**a**) Photo of Zr-W interface, (**b**,**c**) distribution of W and Zr elements at the interface, (**d**) selected electron diffraction results of the intermediate diffusion layer, and (**e**) diffraction fringes of the diffusion layer.

Figure 5 presents the cross-sectional microstructural evolution of HIP-1 specimen prepared by focused ion beam (FIB). Figure 5a displays a bright-field TEM micrograph of the W-Zr alloy cross-section, revealing coarse equiaxed W grains. Figure 5b,c show the elemental distribution of Zr and W at the interface; it can be clearly seen that Zr and W atoms undergo sufficient diffusion at the interface, and a clear diffusion layer is formed in the middle. Figure 5d,e present selected area electron diffraction results and high-resolution images of the E region in the diffusion layer. The ring-type pattern confirms the polycrystalline nature of the diffusion layer; the calibration results of the diffraction ring indicate that the phase of the diffusion layer is ZrW_2_. Analysis of the Moir é fringes in the high-resolution image shows an interplanar spacing of 0.62 nm, corresponding to either (003) or (022) crystallographic planes of ZrW_2_.

### Mechanical Properties of W-Zr Alloy Interface

**Figure 6:**
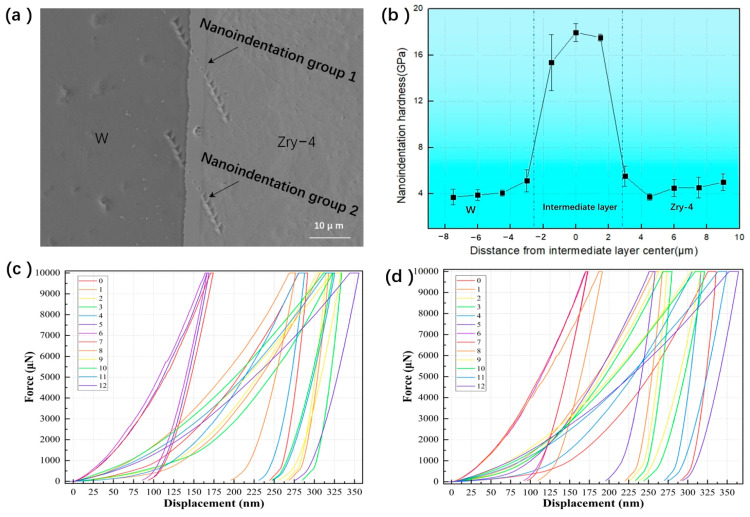
Nanoindentation hardness results of HIP-4 sample interface. (**a**) Morphology of sample interface after nanoindentation, (**b**) nanoindentation hardness curve of sample interface, (**c**) Indentation depth and force curve of each point in group 1, (**d**) Indentation depth and force curve of each point in group 1, different colors represent different nanoindentation points.

Figure 6a–d show the nanoindentation hardness measurement results across the interface region of the HIP-4 sample, with the left side representing W and the right side Zry-4. Figure 6a shows the morphology of the sample after the nanoindentation hardness test, where two tests series were performed at the interface, with 11 points in each group. Four points were tested on the W, five points were tested on the Zry-4, and three points were tested on the interface diffusion layer. Figure 6c,d show the load–displacement curves for nanoindentation group 1 and group 2 under a maximum of 10 mN. These curves demonstrate the expected positive correlation between applied force and penetration depth. Due to the fixed force value used in this experiment, different indentation depths are reflected for different areas of the interface due to different hardness; the depth of the nanoindentation is between 160 nm and 360 nm. Figure 6b shows the nanoindentation hardness values at each point; the horizontal axis represents the distance from the point to the center of the diffusion layer. The data clearly reveal that the intermediate diffusion layer exhibits higher nanoindentation hardness compared to both W and Zry-4 matrix, reaching a maximum of 17.96 GPa. Combined with TEM characterization, these mechanical property measurements confirm that the ZrW_2_ phase formed in the diffusion layer behaves as a typical brittle intermetallic compound.

**Figure 7:**
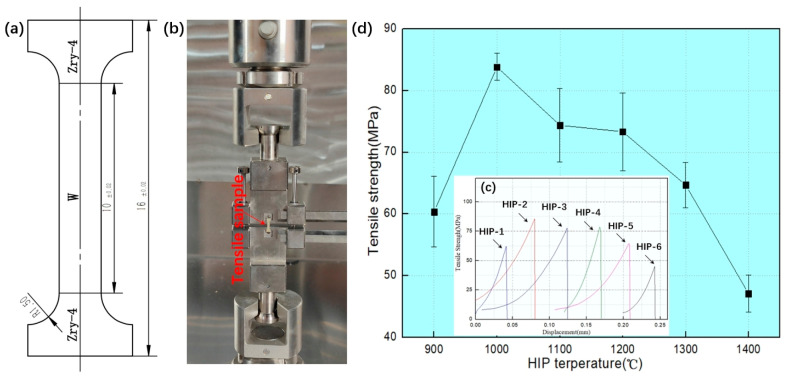
The bonding strength of Zry-4–W composite. (**a**) Schematic diagram of tensile sample size of W-Zr welded joint, (**b**) tensile test photo of W-Zry-4 composite samples, (**c**) tensile strength curves of samples at different HIP temperatures, and (**d**) tensile curves of different samples.

Figure 7a illustrates the specimen geometry for W-Zr welded joint tensile testing. Figure 7b shows the experimental setup of W-Zry-4 composite samples; the sample is installed in a sample holder, and then the sample holder is hung on the universal testing machine for tensile testing. The mechanical performance results are presented in Figure 7c,d. Figure 7c gives the tensile strength curves of the samples at different HIP temperatures. The results show that as the HIP temperature increased from 900 °C to 1400 °C, the bonding strength between the Zry-4 and W first increased and then decreased; the maximum bonding strength is 83.9 MPa when the HIP temperature is 1000 °C. Figure 7d presents complete stress–strain curves, demonstrating consistently brittle fracture behavior across all specimens with negligible plastic deformation prior to failure.

The intermetallic compounds formed at the welding interface significantly influence the mechanical properties [25,26,27]. These brittle intermetallic compounds show a significant difference in terms of properties with the matrix, which is detrimental to the connection strength of the joint. During welding W and Zry-4, at the beginning, W and Zr atoms diffuse to form ZrW_2_ intermetallic compound, which is very thin in thickness; ZrW_2_ serves as a transition layer connecting Zry-4 and W, so the bonding strength increases as the thickness increases. However, excessive growth induces interfacial residual stresses due to property disparities, ultimately reducing joint strength. To optimize joint performance, the formation of intermediate brittle phases in the diffusion layer should be avoided; a good approach is to add a transition metal layer in the middle during welding, as this transition layer metal can form good solid solution with the substrate. For the diffusion welding of W and zirconium alloys, pure metal Ta and Ti are excellent transition metal layers, and the addition of transition metal layers greatly improves welding quality [28].

## 4. Discussion

**Figure 8:**
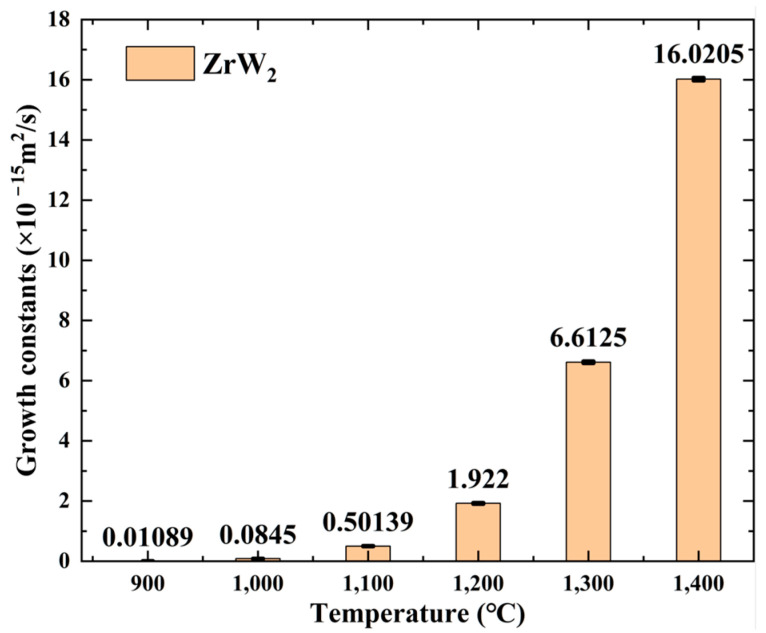
Growth constants of ZrW_2_ (×10^−15^ m^2^/s).

Under high-temperature and high-pressure conditions, an interfacial ZrW_2_ diffusion layer forms through mutual W-Zr diffusion. The growth of the ZrW_2_ phase is controlled by the diffusion mechanism and can be calculated using the parabolic growth constant of the ZrW_2_ phase at different temperatures, combined with Equation (1) [29,30].

(1) $$K_{p}=\frac{x^{2}}{∆t}$$

where $K_{p}$ is the parabolic growth constant ${(m}^{2}/s)$, x is the thickness of the ZrW_2_ phase, and ∆t is the diffusion time. The parabolic growth constant is shown in Figure 8.

**Figure 9:**
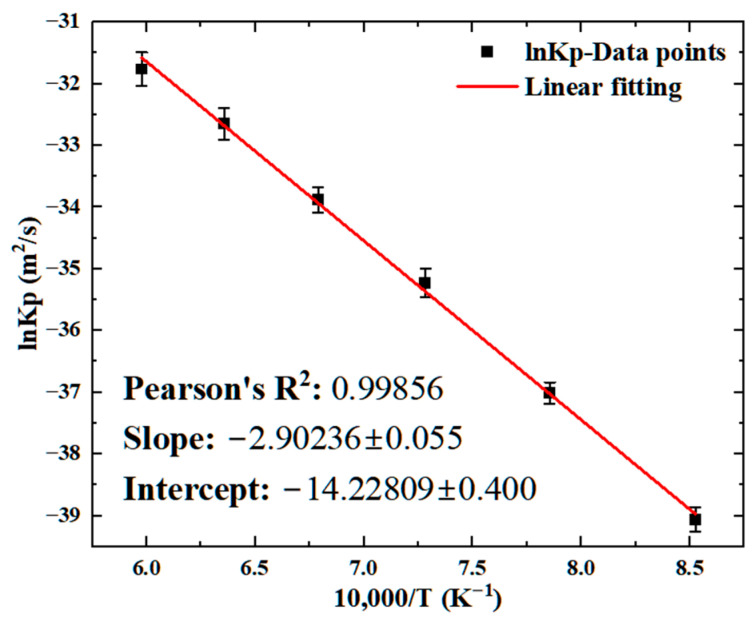
Temperature dependence of growth constants calculated for ZrW_2_.

To quantitatively calculate the activation energy for ZrW_2_ phase growth, the parabolic growth constant $K_{p}$ was logarithmically transformed, resulting in a plot of the growth constant versus temperature, as shown in Figure 9. In this plot, ${lnK}_{p}$ is on the vertical axis, and 10,000/T is on the horizontal axis. The excellent liner fit ($R^{2}$) achieved was 0.9988, indicating that the temperature-dependent growth kinetics obey the Arrhenius relationship [31,32]:

(2) $$lnK_{p}=lnK_{0}-\frac{Q}{RT}$$

where *Q* is the growth activation energy (kJ/mol), *T* is the annealing temperature in Kelvin (K), *R* is the ideal gas constant with a value of 8.314 (J/mol·K), and $K_{0}$ is the pre-exponential factor. The intercept of the fitted line corresponds to ${lnK}_{0}$, and the slope corresponds to −Q/R. The parabolic growth constant $K_{p}$, being proportional to the diffusion coefficient *D*, inherently adopts an Arrhenius-type temperature dependence due to the thermally activated nature of atomic diffusion. As the diffusion coefficient itself follows the Arrhenius equation, the temperature dependence of $K_{p}$ directly inherits this activation-controlled behavior. This fundamental relationship is theoretically derived from the dominance of diffusion-limited kinetics in the growth mechanism. Supported by experimental data from this study, which demonstrates a linear correlation between ln${(K}_{p})$ and *1/T*, the Arrhenius relationship governing $K_{p}(T)$ has been quantitatively validated, with the derived activation energy *Q* aligning closely with literature values for interdiffusion between Zr/W systems.

Based on the fitting results from Figure 9, the pre-exponential factor and growth activation energy for the ZrW2 phase were calculated. The results obtained were Q=241.3022±4.5727 kJ/mol and $K_{0}=6.6194\pm 2.1823\times 10^{-7}\;m^{2}/s$. These results were compared with literature data in Table 3. The measured activation energy for Zr/W interdiffusion in this study (HIP) (241 kJ/mol) is lower than the reported value (285 kJ/mol) obtained via hot pressing technology [32]. This discrepancy can be discussed from the following aspects: (i) Dynamic recrystallization and grain refinement [33,34,35]: The high-pressure HIP environment (100 MPa in this experiment) enhances dynamic recrystallization compared to conventional processing, resulting in finer grains and higher grain boundary density. A higher grain boundary density facilitates atomic diffusion through enhanced short-circuit pathways along grain boundaries, thereby resulting in a lower measured activation energy for diffusion. (ii) Stress-assisted diffusion effects [36,37]: Compared to hot pressing, HIP generates higher and more uniform compressive stresses at the Zr/W interface. This high-pressure condition promotes localized lattice distortion and plastic deformation, forming dislocation networks that serve as accelerated diffusion pathways. Such stress-induced microstructural changes effectively lower the atomic diffusion energy barrier, explaining the reduced activation energy observed in HIP. (iii) Guidance for HIP process optimization: The measured lower activation energy indicated that the Zr/W interdiffusion rate under HIP conditions is approximately one order of magnitude higher than in hot pressing at equivalent temperatures. These enhanced diffusion kinetics enabled two strategic optimization strategies: (1) achieving comparable diffusion effects at reduced processing temperatures or (2) maintaining identical temperatures while significantly shortening dwell times. Both approaches improve manufacturing efficiency without compromising interfacial bonding quality. This systematic comparison highlights the fundamental advantages of HIP technology in controlling interfacial diffusion behavior through its unique pressure-assisted mechanisms, providing theoretical guidance for optimizing diffusion-sensitive joining processes.

While the growth activation energy values obtained in this work show discrepancies with previous reports, the main reasons for these differences include (i) substantial differences in preparation methods and processing parameters, (ii) differences in the chemical composition of the raw materials, and (iii) experimental measurement uncertainties. Overall, these differences are within an acceptable range. As is well known, the driving force for diffusion is the chemical potential gradient. During the initial stage of diffusion, Zr and W form the intermetallic compound ZrW_2_. As diffusion progresses, a (Zr, W) solid solution forms between Zr and ZrW_2_, and later, ZrW_2_ forms between the (Zr, W) solid solution and pure W. In summary, during the diffusion process, a (Zr, W) solid solution forms between ZrW_2_ and Zr, leading to a reduction in the chemical potential for the formation of ZrW_2_.

Generally speaking, in the diffusion bonding of dissimilar metals, the intermetallic compounds formed at the interface will affect the performance of the joint, resulting in a decrease in joint strength. In this study, the ZrW_2_ phase formed at the interface is an intermetallic compound with significantly higher hardness than both the W and Zry-4 alloy matrix. To improve welding quality, it is necessary to avoid the formation of such brittle phases as much as possible during welding. A potential mitigation strategy involves inserting a slow-release layer in the middle, such as Ta, which has been reported in relevant articles [28].

## 5. Conclusions

This study investigates the fabrication of W-Zry4 layered composites using HIP diffusion welding across a temperature ranging from 900 °C to 1400 °C. Nanoindentation testing, tensile strength testing, microstructure observation, and phase structure analysis were conducted on the W/Zry-4 interface. A summary of our findings is as follows:

(1) Within the 900 °C to 1400 °C diffusion welding temperature range, W and Zr joints develop metallurgical bonding with a distinct interfacial layer. The intermediate layer thickness increases from 0.28 μm to 10.74 μm with a rising HIP temperature.
(2) ZrW_2_ intermetallic phase was formed at the W/Zry-4 interface after HIP diffusion welding.
(3) The ZrW_2_ intermediate layer exhibits a hardness of 17.96 GPa, which is significantly higher than that of base materials Zry-4 and W.
(4) The W-Zr interfacial bonding strength demonstrates a non-monotonic temperature dependence: (i) it increases initially with temperature, (ii) peaks at 83.9 MPa at 1000 °C HIP temperature, and (iii) subsequently decreases at higher temperatures.

## Figures and Tables

**Table 1:** Preparation process parameters and dimensions of 5 samples.

Samples ID	HIP Parameters		
	Temperature	Holding Time	Pressure
HIP-1	900 °C	2 h	100 MPa
HIP-2	1000 °C	2 h	100 MPa
HIP-3	1100 °C	2 h	100 MPa
HIP-4	1200 °C	2 h	100 MPa
HIP-5	1300 °C	2 h	100 MPa
HIP-6	1400 °C	2 h	100 MPa

**Table 2:** Thickness of interface diffusion layer for samples at different HIP temperatures.

Specimens ID	HIP Temperature (°C)	Intermediate Layer Thickness (μm)
HIP-1	900 °C	0.28
HIP-2	1000 °C	0.78
HIP-3	1100 °C	1.90
HIP-4	1200 °C	3.72
HIP-5	1300 °C	6.90
HIP-6	1400 °C	10.74

**Table 3:** Calculated $K_{p}$ values for different specimens.

Specimens ID	T (°C)	x (μm)	$K_{p}{(\times 10^{-15}m}^{2}/s)$
HIP-1	900	0.28	0.01089
HIP-2	1000	0.84	0.0845
HIP-3	1100	1.90	0.50139
HIP-4	1200	3.75	1.922
HIP-5	1300	6.75	6.6125
HIP-6	1400	10.75	16.0205
$K_{0}(\times 10^{-7}m^{2}/s)$	6.6194061±2.18229 (This work)		
Q(kJ/mol)	241.3022±4.5727 (This work)		285 [32]

## Data Availability

The original contributions presented in this study are included in the article. Further inquiries can be directed to the corresponding authors.
